# Predicting Ecological Risks of *Alexandrium* spp. Under Climate Change: An Ensemble Modeling Approach

**DOI:** 10.3390/biology14111499

**Published:** 2025-10-27

**Authors:** Ru Lan, Luning Li, Rongchang Chen, Yi Huang, Cong Zhao, Nini Wang

**Affiliations:** 1 China Waterborne Transport Research Institute, Beijing 100088, China; lanru@wti.ac.cn (R.L.); liluning@wti.ac.cn (L.L.); chenrongchang@wti.ac.cn (R.C.); 2 School of Energy and Environmental Engineering, University of Science and Technology Beijing, Beijing 100083, China; 3 Key Laboratory of Biodiversity and Environment on the Qinghai-Tibetan Plateau, Ministry of Education, School of Ecology and Environment, Tibet University, Lhasa 850000, China; hyhy1232021@163.com; 4 School of Environmental Science and Engineering, Southwest Jiaotong University, Chengdu 611756, China; zhaocongmy@my.swjtu.edu.cn

**Keywords:** *Alexandrium* spp., ensemble modeling, species distribution model, potential distribution, ecological risk management

## Abstract

**Simple Summary:**

Harmful algal blooms (HABs) caused by *Alexandrium* spp. are a growing threat to marine ecosystems, fisheries, and human health. This study used computer-based ensemble models to predict where these algae might occur in Chinese coastal waters now and in the future under climate change. We found that salinity is the most important factor controlling their presence, while temperature influences when and where blooms may happen. At present, the highest-risk areas are in Bohai Bay, the Yangtze River estuary, and along the Fujian and Guangdong coasts. In the future, suitable habitats are expected to shift southward, especially under high-emission scenarios, with new risk zones emerging in the South China Sea. These findings suggest that climate change will restructure regional bloom risks and highlight the need for proactive monitoring and management to protect fisheries and public health.

**Abstract:**

*Alexandrium* spp., globally recognized as harmful algal bloom (HAB) species, pose severe threats to marine ecosystems, fisheries, and public health. Based on 469 occurrence records and 24 marine environmental variables, this study employed the Biomod2 ensemble modeling framework to predict the potential distribution of *Alexandrium* spp. under current and future climate scenarios, and to assess the role of key environmental factors and the spatiotemporal dynamics of habitat centroid shifts. The results revealed that (1) the ensemble model outperformed single models (AUC = 0.998, TSS = 0.977, Kappa = 0.978), providing higher robustness and reliability in prediction; (2) salinity range (bio18, 19.1%) and mean salinity (bio16, 5.8%) were the dominant factors, while minimum temperature (bio23) also showed strong constraints, indicating that salinity determines “whether persistence is possible,” while temperature influences “whether blooms occur”; (3) under present conditions, high-suitability habitats are concentrated in Bohai Bay, the Yangtze River estuary to the Fujian coast, and parts of Guangdong; (4) climate change is predicted to drive a southward shift of suitable habitats, with the most pronounced expansion under the high-emission scenario (RCP8.5), leading to the emergence of new high-risk areas in the South China coast and adjacent South China Sea; (5) centroid analysis further indicated a pronounced southward migration under RCP8.5 by 2100, highlighting a regional reconfiguration of ecological risks. Collectively, salinity and temperature are identified as the core drivers shaping the ecological niche of *Alexandrium* spp., and future warming is likely to exacerbate HAB risks in southern China. This study delineates key prevention regions and proposes a shift from reactive to proactive management strategies, providing scientific support for HAB monitoring and marine ecological security in China’s coastal waters.

## 1. Introduction

Global climate change and intensified human activities are profoundly reshaping the structure and function of marine ecosystems. Among the emerging ecological threats, the outbreaks of invasive harmful algal blooms (HABs) have become a pressing issue for environmental security and public health [1,2]. In recent decades, alien algal species introduced through ballast water, aquaculture practices, and global maritime trade have been increasingly reported in China’s coastal waters. Their spread and colonization are often accompanied by ecological disasters such as HABs, which not only cause severe losses to fisheries but also threaten human health through bioaccumulation along food chains [2,3,4]. Against this background, identifying the potential distribution of typical HAB species and understanding their responses to climate change are of both scientific and practical significance for marine bioinvasion control and ecological security management.

Within the diverse HAB taxa, *Alexandrium* spp. have attracted particular attention due to their production of paralytic shellfish toxins. These dinoflagellates are widely distributed in global coastal regions, and their population expansion and bloom events are frequently associated with large-scale HAB outbreaks [5,6]. Such events can cause mass mortality of bivalves and fish, disrupt mariculture, and impose serious risks on fisheries and public health [7,8]. Previous studies have shown that *Alexandrium* spp. exhibit strong environmental tolerance. Their resting cysts can survive for long periods under unfavorable conditions and germinate rapidly when temperature, salinity, and nutrient levels become favorable [9,10,11]. This adaptive strategy enables them to establish stable populations in new environments and subsequently trigger bloom events. Therefore, exploring the ecological niche distribution of *Alexandrium* spp. and its spatiotemporal dynamics under climate change is essential for predicting and managing invasion risks.

Species distribution models (SDMs) have become widely used tools for investigating the spatial patterns of HABs. By linking species occurrence records with environmental variables, SDMs can identify suitable habitats and project potential range shifts under future climate scenarios [12,13]. Among these models, the maximum entropy model (MaxEnt) has been widely applied in HAB studies due to its low data requirements. However, single-model approaches are often limited by high prediction uncertainty and strong dependence on specific algorithms [14,15]. In recent years, ensemble modeling has emerged as a more robust framework. The Biomod2 platform, developed in R, integrates multiple algorithms and improves stability and accuracy through weighted ensemble predictions [16,17]. By excluding poorly performing models and combining stronger ones, Biomod2 reduces uncertainty among models and provides a more realistic representation of potential distributions [18].

Although the ecology and distribution of *Alexandrium* spp. have been studied, systematic predictions based on ensemble modeling frameworks remain scarce. In particular, few studies have quantitatively identified the dominant environmental factors, assessed future habitat expansion or contraction, and tracked the migration of habitat centroids under climate change. To address these gaps, this study focused on four major seas of China (Bohai Sea, Yellow Sea, East China Sea, and South China Sea). Using actual occurrence records and key environmental variables, we applied the Biomod2 framework to model the potential distribution of *Alexandrium* spp. under present and future climate scenarios. The objectives were to (1) identify the main environmental factors shaping the distribution of *Alexandrium* spp. and evaluate their relative importance; (2) predict the potential distribution and variation trends under different climate scenarios using ensemble modeling; and (3) analyze the spatiotemporal dynamics of centroid shifts and regional differences under climate change. The results provide new insights into the ecological adaptation mechanisms of HAB species and scientific support for risk assessment and coastal management.

## 2. Materials and Methods

### 2.1. Species Distribution Records

This study focused on *Alexandrium* spp., one of the most representative harmful algal bloom (HAB) genera. Species distribution data were obtained from systematic sampling and analysis of ballast tank sediments from international vessels docking at major Chinese ports [19]. Resting cysts and germinated vegetative cells of *Alexandrium* spp. were identified through morphological methods (light microscopy and scanning electron microscopy) and molecular techniques, including sequencing of the large subunit ribosomal RNA (LSU) and internal transcribed spacer (ITS) regions. A total of 469 precise geographic occurrence sites were recorded [19]. Geographic coordinates were collected using a global positioning system (GPS). To minimize spatial autocorrelation and avoid model overfitting, occurrence points were thinned using the spThin package in R, ensuring a minimum distance of 10 km between records.

### 2.2. Environmental Variables

**Figure 1:**
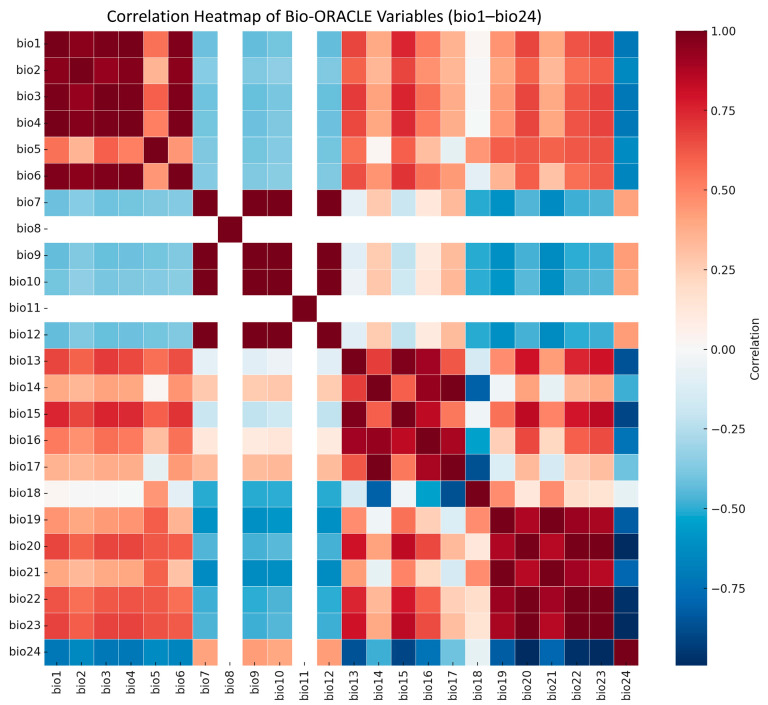
Pearson correlation heatmap of 24 candidate environmental variables. The color gradient indicates the correlation coefficient (r), with red representing positive correlations and blue representing negative correlations.

We initially collected 24 environmental variables describing marine hydrological and climatic conditions, including sea surface temperature, salinity, current velocity, and ice thickness (Figure 1 and Table 1; see detailed variable definitions in Table S1). These 24 variables were selected to comprehensively represent thermal, saline, hydrodynamic, and ice-related gradients that influence *Alexandrium* ecology, following standard practices in marine species-distribution modeling. Environmental data layers were obtained from the Bio-ORACLE v2.1 database (https://www.bio-oracle.org) and the World Ocean Atlas 2018 dataset (https://www.ncei.noaa.gov/products/world-ocean-atlas, accessed on 1 August 2023), both of which provide long-term climatological means of marine environmental parameters at a 2.5 arc-minute spatial resolution. Present-day regional observations for Chinese coastal waters were additionally referenced from the China Marine Environment Monitoring Network (http://www.cnemc.cn/) to ensure local representativeness. Future climate projections were derived from the Intergovernmental Panel on Climate Change (IPCC) Fifth Assessment Report (AR5) under three Representative Concentration Pathways (RCPs): RCP2.6, RCP4.5, and RCP8.5, representing low-, medium-, and high-emission scenarios, respectively.

**Figure 2:**
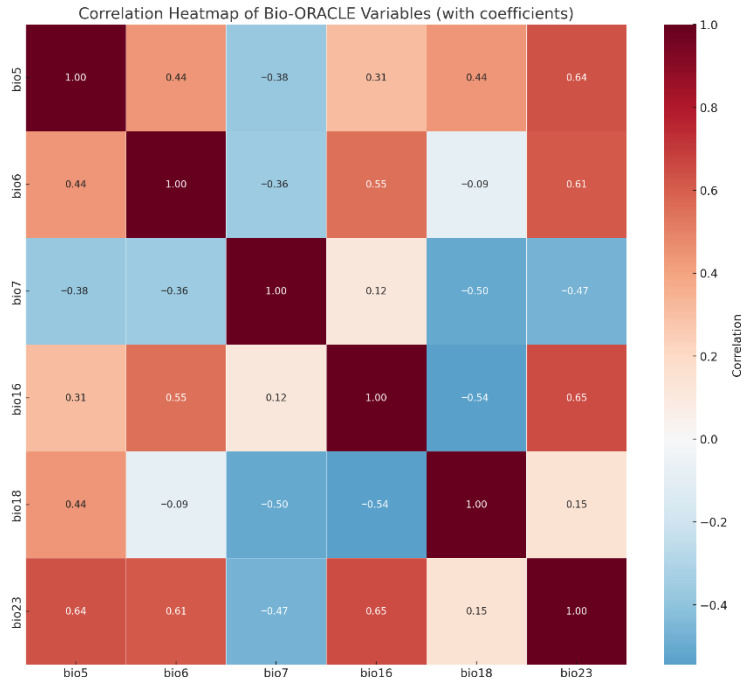
Pearson correlation heatmap of six key environmental variables (bio5: minimum current velocity, bio6: current velocity range, bio7: maximum ice thickness, bio16: mean salinity, bio18: salinity range, bio23: minimum temperature).

To reduce multicollinearity among variables, we performed Pearson correlation analysis (Figure 2). When the correlation coefficient exceeded |r| ≥ 0.8, only the variable with greater ecological relevance was retained [20]. After screening, six key variables were selected for modeling: minimum current velocity (bio5), current velocity range (bio6), maximum ice thickness (bio7), mean salinity (bio16), salinity range (bio18), and minimum temperature (bio23) (Table 1). These predictors captured the main hydrological and climatic gradients relevant to *Alexandrium* spp. distribution.

### 2.3. Ensemble Model Construction

The potential distribution of *Alexandrium* spp. was modeled using the Biomod2 package (version 4.2-5) in R 4.3.2. Nine algorithms were integrated [21]: generalized linear model (GLM), generalized additive model (GAM), generalized boosted model (GBM), classification tree analysis (CTA), artificial neural network (ANN), surface range envelope (SRE), multivariate adaptive regression splines (MARS), random forest (RF), and maximum entropy (MaxEnt). For background sampling, 1000 pseudo-absence points were randomly generated across the study area and weighted equally with occurrence records to avoid bias.

Model parameters were optimized using the biomod-tuning function. Data were split into training (75%) and testing (25%) sets, and each model was replicated 10 times to reduce stochastic effects, producing 90 single-model runs [22,23]. Model performance was evaluated with three metrics: the Kappa statistic, the true skill statistic (TSS), and the area under the receiver operating characteristic curve (AUC). Only models with AUC ≥ 0.8 were retained for ensemble building. Final ensemble predictions were generated using weighted means of qualified models, thereby enhancing stability and reliability [24,25].

Predicted probabilities were converted into habitat suitability categories based on threshold values: unsuitable (0.0–0.25), low (0.25–0.5), moderate (0.5–0.8), and high suitability (0.8–1.0). Spatial visualization and mapping were conducted in ArcGIS v10.4.1, providing the basis for subsequent analyses of habitat suitability and centroid shifts.

### 2.4. Ecological Niche Analysis

To evaluate potential niche shifts under climate change, we applied the ecospat package (v4.1.1) in R [26]. Current and future suitability maps were binarized into suitable/unsuitable categories, and Schoener’s D index was used to quantify niche overlap. Niche equivalency and similarity tests were further conducted to assess the statistical significance of niche shifts across climate scenarios.

### 2.5. Centroid Migration Analysis

Habitat centroid migration was quantified using the SDMTools package (v1.1-21) in R. For each climate scenario and time period [26], the centroid coordinates of suitable habitats were calculated and mapped in ArcGIS v10.4.1. Distances and directions of centroid shifts were measured, enabling visualization of the spatiotemporal dynamics of *Alexandrium* spp. distribution under climate change.

## 3. Results

### 3.1. Model Accuracy

**Figure 3:**
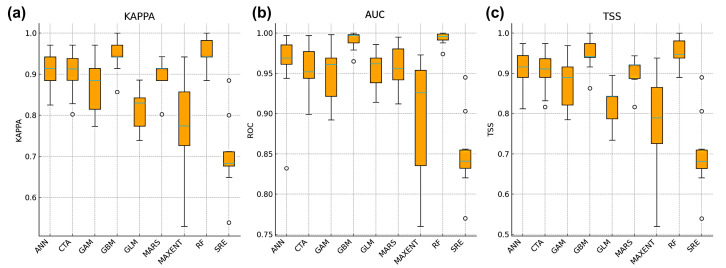
Comparison of prediction accuracy among different distribution models based on 10 replicated runs. (**a**) Kappa statistics, (**b**) area under the receiver operating characteristic curve (AUC), and (**c**) true skill statistic (TSS) were used to evaluate model performance.

To compare the predictive performance of single models and the ensemble model, we evaluated accuracy using the Kappa, AUC, and TSS indices (Figure 3; Table 2). Most single models, including GBM, RF, MARS, GLM, and MaxEnt, achieved high and stable accuracy, with RF and GBM showing the strongest discriminative ability (AUC and TSS values approaching 1.0). CTA and ANN also performed well, with only slightly lower median scores compared with the top-performing models. In contrast, SRE exhibited markedly lower accuracy and greater variability across replicates, indicating its limited predictive robustness.

The ensemble model (EMwmean) outperformed all individual algorithms. Its Kappa, AUC, and TSS values were consistently higher and showed less variation, reflecting improved stability and reliability. AUC values across all algorithms remained high, suggesting that the model effectively distinguished suitable from unsuitable habitats [27]. Overall, ensemble modeling successfully integrated the strengths of multiple algorithms and reduced biases inherent in single models, providing a robust basis for subsequent distribution predictions.

### 3.2. Environmental Drivers of Distribution

**Figure 4:**
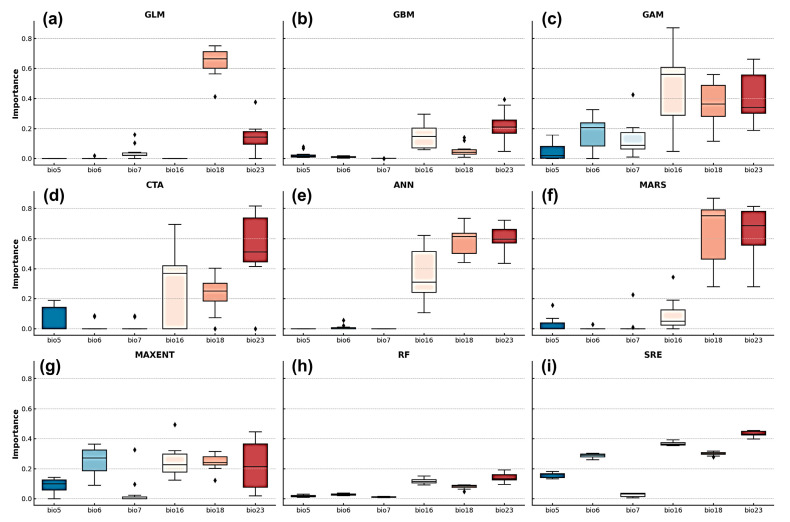
Distribution of variable importance for six key environmental factors (bio5–bio23) across nine single models (GLM, GBM, GAM, CTA, ANN, MARS, MAXENT, RF, and SRE). Panels (**a**–**i**) are arranged from left to right and top to bottom. Boxplots show contribution values from 10 replicate runs, with the line inside each box indicating the median, box edges the interquartile range, and circles outliers.

Variable-importance analyses from the nine single models (Figure 4) revealed consistent patterns across algorithms. Salinity-related factors dominated the predictions, salinity range (bio18) and mean salinity (bio16) exhibited the highest importance in most models, followed by minimum temperature (bio23), indicating that salinity gradients and temperature jointly determine habitat suitability. Current-velocity variables (bio5 and bio6) showed relatively low but consistent contributions across algorithms such as GAM, MAXENT, and SRE, suggesting a persistent yet secondary influence of hydrodynamic exchange and water-mass movement on *Alexandrium* spp. dispersal. In contrast, ice thickness (bio7) displayed lower and more variable responses, contributing noticeably only in a few models (e.g., CTA, GAM), implying that ice-cover effects are spatially limited under current climatic conditions.

**Figure 5:**
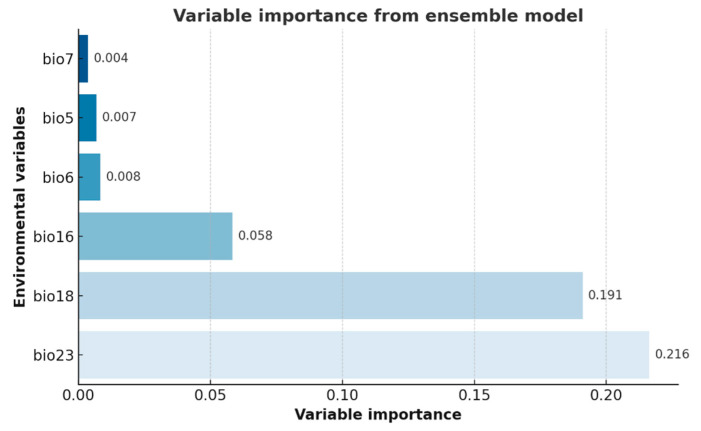
Relative importance of six key environmental factors in the ensemble model.

The ensemble model (Figure 5) synthesized these single-model outputs into a weighted average, yielding a more stable and representative assessment of predictor influence. It confirmed the predominant role of salinity, with the combined contribution of salinity range (bio18, 19.1%) and mean salinity (bio16, 5.8%) reaching 24.9%, slightly exceeding that of minimum temperature (bio23, 21.6%). This cumulative dominance indicates that overall salinity gradients exert a stronger influence on habitat suitability than individual thermal or hydrodynamic factors, while ice cover contributed least. Collectively, Figure 4 and Figure 5 illustrate that the ensemble results consolidate the patterns observed in individual models, highlighting salinity, temperature, and current velocity as the primary environmental drivers governing *Alexandrium* spp. distribution.

### 3.3. Potential Geographical Distribution of Alexandrium spp. Under Current Climatic Conditions

**Figure 6:**
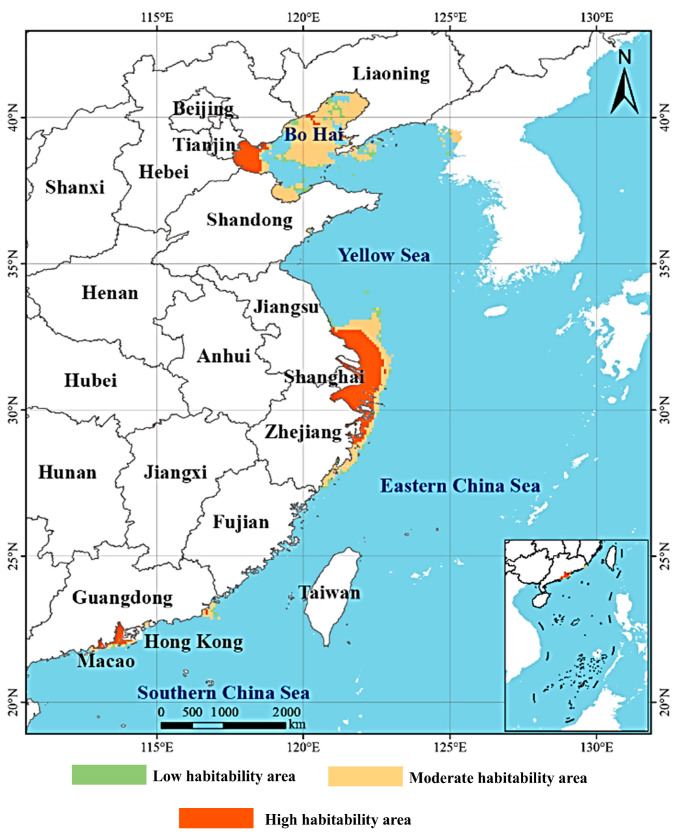
Potential habitat distribution of *Alexandrium* spp. under current climate conditions. Habitat suitability is categorized into low (green), moderate (yellow), and high (orange) classes.

Under present climatic conditions, the ensemble model predicted that *Alexandrium* spp. habitats are concentrated along China’s eastern coast and several nearshore areas (Figure 6). Suitability patterns showed a patchy yet clustered distribution, with high suitability in Bohai Bay, the northern Yellow Sea, the Yangtze River estuary, and coastal Fujian. These regions share characteristics such as stable salinity, weak hydrodynamics, and nutrient enrichment, providing favorable conditions for *Alexandrium* spp. persistence and bloom development [28,29].

The total suitable habitat area was estimated at 17.61 × 10^4^ km^2^, comprising 1.44 × 10^4^ km^2^ of low suitability, 8.95 × 10^4^ km^2^ of moderate suitability, and 7.22 × 10^4^ km^2^ of high suitability. The large proportion of moderate and high suitability zones suggests strong potential for spread and colonization, especially along the Yangtze estuary to Fujian, where a continuous band of suitable habitat was identified. Estuaries and semi-enclosed bays such as Bohai Bay and Hangzhou Bay also showed medium-to-high suitability, reflecting their limited water exchange and frequent nutrient input [30,31]. In contrast, offshore waters with strong currents and high salinity variability were generally unsuitable, showing only scattered patches of low suitability.

### 3.4. Prediction of the Impact of Climate Change on the Potential Geographic Distribution of Alexandrium spp.

**Figure 7:**
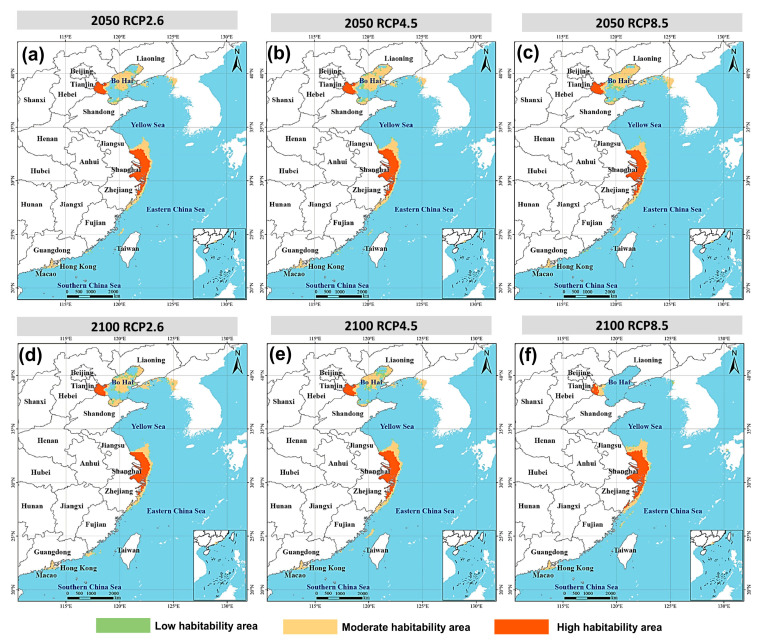
Predicted potential distribution of *Alexandrium* spp. under different climate change scenarios. Panels (**a**–**c**) represent projections for 2025 under RCP2.6, RCP4.5, and RCP8.5, respectively; panels (**d**–**f**) represent projections for 2100 under RCP2.6, RCP4.5, and RCP8.5, respectively. Habitat suitability is categorized into low (green), moderate (yellow), and high (orange) classes.

Predicted habitat patterns under future climate scenarios showed strong temporal and spatial dynamics (Figure 7; Table 3 and Table 4). Low-suitability areas remained relatively stable, while moderate- and high-suitability areas shifted significantly among scenarios.

By 2050, under RCP2.6, moderate and high suitability areas increased slightly to 9.27 × 10^4^ km^2^ and 7.11 × 10^4^ km^2^, maintaining overall stability. Under RCP4.5 and RCP8.5, moderate suitability areas expanded markedly (10.46 × 10^4^ km^2^ and 10.62 × 10^4^ km^2^, respectively), while high suitability areas remained similar (7.21 × 10^4^ km^2^ and 6.92 × 10^4^ km^2^). These results indicate enhanced suitability around the Yangtze estuary and Fujian coast under stronger emission scenarios.

By 2100, divergence among scenarios became more pronounced. Under RCP2.6, moderate (9.98 × 10^4^ km^2^) and high suitability areas (6.59 × 10^4^ km^2^) remained relatively stable. Under RCP4.5, high suitability areas increased (7.34 × 10^4^ km^2^) while moderate areas declined (8.57 × 10^4^ km^2^), reflecting structural adjustments within suitable zones. Under RCP8.5, contraction dominated: moderate suitability shrank sharply to 5.29 × 10^4^ km^2^, while high suitability persisted (7.33 × 10^4^ km^2^). This scenario suggested a southward concentration of suitable habitats, with reduced stability in northern waters.

Expansion and contraction dynamics confirmed these patterns (Table 4). In 2050, all scenarios showed greater expansion than contraction, indicating overall habitat growth. However, by 2100 under RCP8.5, contraction reached 7.25 × 10^4^ km^2^, surpassing expansion (2.92 × 10^4^ km^2^) and leaving only 10.36 × 10^4^ km^2^ unchanged. This highlighted large-scale habitat restructuring under extreme climate conditions. Spatially, suitable habitats clustered along the Yangtze–Fujian coast and extended southward into Guangdong and the South China Sea, while Bohai and the Yellow Sea gradually declined in suitability.

### 3.5. Centroid Shift Analysis of Potential Suitable Habitats

**Figure 8:**
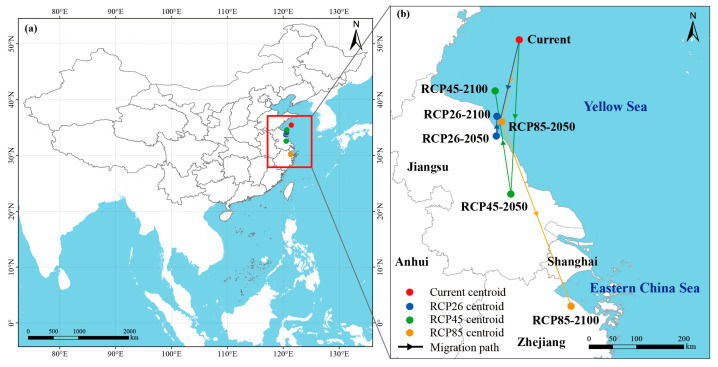
Migration trajectories of the potential habitat centroid of *Alexandrium* spp. under current and future climate scenarios. (**a**) Study area location; (**b**) centroid migration paths. The red dot represents the current centroid, while blue, green, and orange dots indicate predicted centroids in 2050 and 2100 under RCP2.6, RCP4.5, and RCP8.5 scenarios, respectively. Arrows show the migration direction.

Centroid analysis further illustrated distributional dynamics (Figure 8). Under present conditions, the centroid was located near the Yangtze River estuary. By 2050, centroids under RCP2.6 and RCP4.5 shifted only slightly within the northern East China Sea, indicating relative stability. Under RCP8.5, however, the centroid moved southward toward coastal Fujian. By 2100, RCP2.6 and RCP4.5 centroids remained near the Yangtze estuary and East China Sea, but RCP8.5 showed a marked southward shift into the South China coast and even toward the northern South China Sea. These patterns suggest stable habitats under low-emission scenarios but strong directional shifts under high emissions, pointing to a climate-driven reconfiguration of *Alexandrium* spp. risk zones.

## 4. Discussion

### 4.1. Accuracy of Ensemble Modeling and Reliability of Niche Prediction

Species distribution models (SDMs) are inevitably influenced by sample size, variable selection, and algorithmic differences, leading to uncertainties in predictions [32]. Single models often have structural biases. For example, generalized linear models (GLMs) are constrained by linear assumptions, artificial neural networks (ANNs) may suffer from gradient vanishing, and surface range envelope (SRE) models lack adaptability under complex conditions [33,34,35]. In this study, while some single models such as RF, MaxEnt, and GBM achieved high accuracy, ANN and SRE performed poorly, failing to capture the full distributional patterns of *Alexandrium* spp.

In contrast, the ensemble model (EMwmean) integrated multiple algorithms through weighted averaging and successfully mitigated the weaknesses of individual models. Its consistently high values of Kappa, AUC, and TSS indicate both numerical precision and predictive stability. This finding is consistent with earlier studies [10,36], which emphasized that ensemble models reduce overfitting and improve reliability. Furthermore, the predicted distributions closely matched known *Alexandrium* spp. habitats, confirming that the ensemble model effectively captured species–environment relationships. Previous HAB studies [36,37] similarly reported that ensemble frameworks provide more robust and interpretable predictions than single models. Therefore, our results highlight ensemble modeling as a reliable approach for assessing future ecological risks under climate change.

### 4.2. Salinity-Driven Niche Constraints

The importance of salinity in shaping *Alexandrium* spp. distribution was striking. Salinity range (bio18) contributed 19.1% and mean salinity (bio16) contributed 5.8%, far exceeding most other predictors. This indicates that salinity not only governs whether populations can persist but also influences bloom intensity and variability. Moderate and stable salinity levels support osmoregulation and metabolic activity in dinoflagellates, while large fluctuations impose physiological stress and suppress long-term persistence [11,38]. Thus, salinity stability emerges as a key determinant of core habitats.

Temperature, especially minimum temperature (bio23), also exerted strong constraints at distributional boundaries. Low temperatures inhibit physiological activity and toxin production, while warming enhances growth and toxicity, increasing bloom potential [39]. Consequently, temperature extremes determine not only the northern limit of distribution but also the timing and intensity of bloom events.

Hydrodynamic conditions contributed moderately. Minimum current velocity (bio5) and velocity range (bio6) revealed that stagnant waters promote nutrient and cell accumulation, favoring blooms, while moderate fluctuations facilitate cyst resuspension and long-term persistence [4]. Excessive turbulence, however, disrupts water stability and inhibits population establishment. In contrast, ice thickness (bio7) played little role, consistent with the fact that *Alexandrium* spp. primarily occurs in temperate and subtropical seas where ice cover is limited.

Together, these results indicate that salinity and temperature are the dominant drivers of *Alexandrium* spp. distribution, while hydrodynamics act at local scales. This pattern aligns with global HAB research, underscoring that salinity stability and extreme temperatures must be prioritized in future risk assessments.

### 4.3. Climate-Driven Southward Shift and Regional Risk Restructuring

Future climate projections indicated significant spatial dynamics in *Alexandrium* spp. habitats. Under low emissions (RCP2.6), habitat patterns remained relatively stable, with over 80% of areas unchanged and only minor local expansion or contraction (Table 4). Under moderate emissions (RCP4.5), suitability expanded markedly, particularly along the Yangtze estuary–Fujian coast, forming continuous high-suitability zones. Under high emissions (RCP8.5), restructuring was most dramatic: by 2100, contraction reached 7.25 × 10^4^ km^2^, expansion was only 2.92 × 10^4^ km^2^, and stable areas declined sharply to 10.36 × 10^4^ km^2^.

Centroid analysis confirmed these trends. Under RCP2.6 and RCP4.5, centroids showed minor fluctuations around the East China Sea, suggesting long-term stability of this region as a core habitat. By contrast, RCP8.5 projections revealed a strong southward shift, with the centroid moving toward Guangdong and the northern South China Sea by 2100. This directional shift implies that future warming will drive a reconfiguration of HAB risk, with northern regions becoming less dominant while southern waters emerge as new hotspots.

These findings are consistent with the climate-tracking hypothesis and global HAB studies. Warming increases sea surface temperature, alters salinity gradients, and strengthens stratification, all of which favor HAB development [1]. Xu et al. (2019) similarly predicted increased HAB risk in South China due to combined warming and runoff effects [40]. Meanwhile, northern regions such as Bohai and the Yellow Sea may experience reduced risk as low-temperature constraints weaken but overall suitability declines. Thus, climate change is expected to shift the epicenter of *Alexandrium* spp. risk southward, restructuring ecological threats along China’s coast.

### 4.4. From Reactive Governance to Proactive Prevention

**Figure 9:**
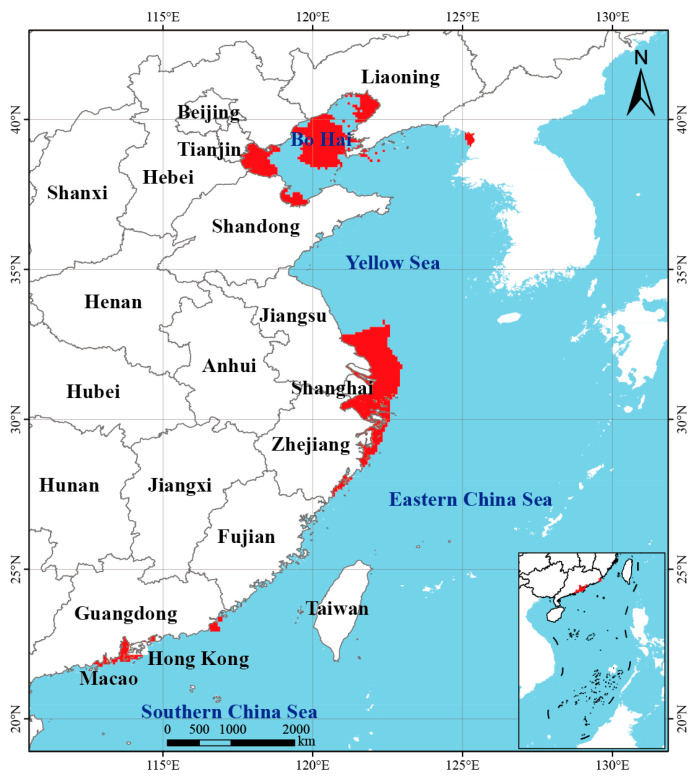
Delineation of key prevention and control areas for *Alexandrium* spp. based on ensemble model predictions. The red regions indicate core high-risk zones with consistently high habitat suitability under both current and future scenarios.

In the context of climate change and habitat restructuring, traditional reactive management of HABs is no longer sufficient. Based on our predictions, three priority prevention regions were identified: (1) Bohai Bay, (2) the Yangtze estuary to Fujian coast, and (3) the Guangdong–South China Sea margin (Figure 9). Among these, the Yangtze–Fujian region remains the core high-risk area under both current and future conditions and should be monitored intensively. Bohai and the northern Yellow Sea, with relatively stable suitability, require continued routine monitoring. Emerging risk zones in Guangdong and the South China Sea should be incorporated into forward-looking management plans.

Globally, HAB management is shifting toward proactive governance based on environmental thresholds. In North America, real-time warning systems combine temperature–salinity thresholds with hydrodynamic models [41]. In Europe, multinational coordination has enhanced HAB risk management [42]. Drawing on these approaches, China should adopt a similar paradigm shift: (i) use salinity, temperature, and hydrodynamics as core indicators in early-warning systems; (ii) implement tiered, region-specific management, prioritizing high-risk zones; and (iii) establish cross-regional monitoring and emergency networks. Such a transition from reactive to proactive management will be essential to mitigate future HAB risks to fisheries, ecosystems, and public health under climate change.

## 5. Conclusions

This study employed an ensemble modeling framework to predict the potential distribution of *Alexandrium* spp. under current and future climate scenarios. The results demonstrated that ensemble modeling substantially outperformed single algorithms in accuracy and stability, making it a reliable approach for ecological niche prediction of harmful algal blooms. Salinity and temperature emerged as the dominant drivers, with salinity stability and minimum temperature jointly constraining distribution boundaries. At present, high-suitability habitats are concentrated in Bohai Bay, the Yangtze River estuary to the Fujian coast, and parts of the Guangdong coast, aligning with known *Alexandrium* spp. hotspots. Future projections suggest that climate change will promote habitat expansion and a pronounced southward shift, particularly under high-emission scenarios where the South China coast and adjacent South China Sea may become new high-risk regions. By delineating key prevention zones and advocating a transition from reactive to proactive management, this study provides a scientific basis for *Alexandrium* spp. monitoring and ecological security in China’s coastal waters.

## Figures and Tables

**Table 1:** 24 environmental variables involved in modeling.

Abbreviation	Environment Variable	Unit
bio1	Maximum monthly mean current velocity	m/s
bio2	Minimum monthly mean current velocity	m/s
bio3	Maximum current velocity	m/s
bio4	Annual mean current velocity	m/s
bio5	Minimum current velocity	m/s
bio6	Annual current velocity range	m/s
bio7	Maximum monthly mean ice thickness	m
bio8	Minimum monthly mean ice thickness	m
bio9	Maximum ice thickness	m
bio10	Annual mean ice thickness	m
bio11	Minimum ice thickness	m
bio12	Annual ice thickness range	m
bio13	Maximum monthly mean salinity	PSU
bio14	Minimum monthly mean salinity	PSU
bio15	Maximum salinity	PSU
bio16	Annual mean salinity	PSU
bio17	Minimum salinity	PSU
bio18	Annual salinity range	PSU
bio19	Maximum monthly mean temperature	°C
bio20	Minimum monthly mean temperature	°C
bio21	Maximum temperature	°C
bio22	Annual mean temperature	°C
bio23	Minimum temperature	°C
bio24	Annual temperature range	°C

**Table 2:** Accuracy evaluation of different distribution models using AUC values, TSS values, and Kappa statistics.

Model	KAPPA_mean	KAPPA_sd	AUC_mean	AUC_sd	TSS_mean	TSS_sd
GLM	0.8168	0.0515	0.9545	0.0229	0.8223	0.0548
GBM	0.9452	0.0391	0.9908	0.0116	0.9460	0.0379
GAM	0.8656	0.0641	0.9428	0.0316	0.8699	0.0602
CTA	0.8997	0.0554	0.9524	0.0292	0.9026	0.0514
ANN	0.9135	0.0434	0.9587	0.0474	0.9146	0.0463
SRE	0.7040	0.0909	0.8501	0.0471	0.6999	0.0945
MARS	0.8969	0.0379	0.9525	0.0273	0.9023	0.0358
RF	0.9483	0.0353	0.9933	0.0079	0.9492	0.0340
MAXENT	0.7691	0.1129	0.8874	0.0720	0.7701	0.1175

_sd represents the standard deviation calculated from 10 replicate runs.

**Table 3:** Predicted area of suitable habitats for *Alexandrium* spp. under future climate scenarios (10^4^ km^2^).

	Low Habitability Area	Moderate Habitability Area	High Habitability Area
present	1.438	8.950	7.222
2050_RCP2.6	1.554	9.266	7.114
2050_RCP4.5	1.596	10.463	7.213
2050_RCP8.5	1.745	10.621	6.923
2100_RCP2.6	1.853	9.981	6.590
2100_RCP4.5	1.803	8.568	7.338
2100_RCP8.5	0.657	5.294	7.330

**Table 4:** Changes in the suitable habitat area of *Alexandrium* spp. under different future climate scenarios (2050 and 2100, RCP26, RCP45, RCP85).

Period	Contraction	Expansion	Unchanged
2050_RCP2.6	2.302	2.626	15.308
2050_RCP4.5	1.321	2.983	16.288
2050_RCP8.5	1.604	3.283	16.006
2100_RCP2.6	1.953	2.767	15.657
2100_RCP4.5	2.726	2.826	14.884
2100_RCP8.5	7.247	2.917	10.363

## Data Availability

The datasets generated and analyzed during the current study are available from the corresponding author on reasonable request.

## Supplementary Materials

The following supporting information can be downloaded at: https://www.mdpi.com/article/10.3390/biology14111499/s1, Table S1. Definition and ecological significance of the 16 marine environmental variables used in this study.
