# Supplementary material for: Predicting Ecological Risks of Alexandrium spp. Under Climate Change: An Ensemble Modeling Approach
Source: Biology (Basel). 2025 Oct 27;14(11):1499. doi: 10.3390/biology14111499 (PMC12650424; doi:10.3390/biology14111499)
Supplement: Supplementary file 1 [file biology-14-01499-s001.zip › biology-3924641-supplementary.pdf]

Supporting materials

Predicting Ecological Risks of *Alexandrium* spp. Under Climate Change: An Ensemble Modeling Approach

Ru Lan [1,2], Luning Li [1], Rongchang Chen [1], Yi Huang [3], Cong Zhao [4] and Nini Wang [1,*]

1 China Waterborne Transport Research Institute, Beijing 100088, China; lanru@wti.ac.cn (R.L.); liluning@wti.ac.cn (L.L.); chenrongchang@wti.ac.cn (R.C.)
2 School of Energy and Environmental Engineering, University of Science and Technology Beijing, Beijing 100083, China
3 Key Laboratory of Biodiversity and Environment on the Qinghai-Tibetan Plateau, Ministry of Education, School of Ecology and Environment, Tibet University, Lhasa 850000, China; hyhy1232021@163.com
4 School of Environmental Science and Engineering, Southwest Jiaotong University, Chengdu 611756, China; zhaocongmy@my.swjtu.edu.cn
* Correspondence: wnn@wti.ac.cn

Table S1. Definition and ecological significance of the 16 marine environmental variables used in this study.

Abbreviation	Environmental variable	Unit	Conceptual definition	Ecological relevance to *Alexandrium* spp.
bio1	Maximum monthly mean current velocity	m s^{-1}	The highest value among the twelve monthly mean current velocities during one year, reflecting the strongest average monthly hydrodynamic condition.	Indicates the upper limit of typical hydrodynamic intensity affecting water column mixing and cyst resuspension over seasonal timescales.
bio2	Minimum monthly mean current velocity	m s^{-1}	The lowest value among the twelve monthly mean current velocities during one year.	Reflects quiescent periods conducive to cell accumulation and bloom formation.
bio3	Maximum current velocity	m s^{-1}	The instantaneous annual peak current velocity observed within the year.	Represents extreme hydrodynamic events (e.g., storms, tides) that may disturb sediments and resuspend cysts.
bio4	Annual mean current velocity	m s^{-1}	The arithmetic mean of all current velocity data throughout the year.	Describes the overall long-term average water-mass movement controlling nutrient transport.
bio5	Minimum current velocity	m s^{-1}	The lowest instantaneous current velocity value recorded within the year.	Indicates potential stagnation periods that allow vertical stratification and bloom persistence.
bio6	Annual current velocity range	m s^{-1}	Difference between the maximum and minimum current velocities in a year.	Measures hydrodynamic variability; large ranges imply unstable environments, influencing cyst germination.
bio7	Maximum monthly mean ice thickness	m	The highest monthly average sea-ice thickness over a year.	Constrains light and temperature regimes in high-latitude seas, indirectly limiting algal growth.
bio8	Minimum	m	The lowest monthly	Indicates seasonal

	monthly mean ice thickness		average sea-ice thickness within a year.	melting conditions affecting water temperature and salinity.
bio9	Maximum ice thickness	m	The instantaneous maximum ice thickness recorded annually.	Represents extreme ice cover events that may completely block photosynthesis.
bio10	Annual mean ice thickness	m	Mean ice thickness throughout the year.	Integrates ice-cover duration and severity, linked to cold-season habitat unsuitability.
bio11	Minimum ice thickness	m	Minimum observed ice thickness during the year.	Reflects open-water periods that permit algal proliferation.
bio12	Annual ice thickness range	m	Difference between maximum and minimum ice thickness in a year.	Describes interseasonal variability of ice cover and the window for bloom development.
bio13	Maximum monthly mean salinity	PSU	The highest mean salinity averaged over any month in a year.	Reflects periods of highest marine intrusion, affecting osmotic tolerance.
bio14	Minimum monthly mean salinity	PSU	The lowest mean salinity averaged over any month in a year.	Indicates freshwater dilution from river discharge or rainfall that may stress algal cells.
bio15	Maximum salinity	PSU	The instantaneous annual salinity peak.	Represents short-term salt-pulse events related to evaporation or tidal forcing.
bio16	Annual mean salinity	PSU	Mean salinity over the entire year.	Describes the background osmotic condition essential for population maintenance.
bio17	Minimum salinity	PSU	The lowest instantaneous salinity observed in a year.	Indicates extreme freshwater influence that may limit cyst viability.
bio18	Annual salinity range	PSU	Difference between maximum and minimum salinity within a year.	Quantifies salinity fluctuation amplitude, a key determinant of habitat stability.
bio19	Maximum	°C	The highest monthly	Reflects summer

	monthly mean temperature		mean sea surface temperature.	thermal maximum affecting growth rate and toxin synthesis.
bio20	Minimum monthly mean temperature	°C	The lowest monthly mean temperature.	Represents winter thermal minimum governing survival limits.
bio21	Maximum temperature	°C	The instantaneous annual maximum temperature.	Captures short-term heat-wave events that may trigger bloom onset.
bio22	Annual mean temperature	°C	Mean annual sea surface temperature.	Determines long-term thermal regime for species persistence.
bio23	Minimum temperature	°C	The lowest instantaneous sea surface temperature recorded in a year.	Defines the cold-tolerance threshold limiting northern distribution.
bio24	Annual temperature range	°C	Difference between annual maximum and minimum temperatures.	Indicates thermal variability affecting metabolic plasticity.

Note: All variables were extracted from the Bio-ORACLE v2.1 marine environmental database at 2.5-arc-minute spatial resolution.
